# Electromagnetic-guided versus endoscopic placement of post-pyloric feeding tubes: a systematic review and meta-analysis of randomised controlled trials

**DOI:** 10.1186/s40560-020-00506-8

**Published:** 2020-12-10

**Authors:** Yaping Wei, Zheng Jin, Ying Zhu, Wei Hu

**Affiliations:** Affiliated Hangzhou First People’s Hospital, Zhejiang University School of Medicine, Hangzhou, China

**Keywords:** Electromagnetic, Endoscopy, Post-pyloric feeding tube, Enteral nutrition

## Abstract

**Background:**

Current evidence supporting the utility of electromagnetic (EM)-guided method as the preferred technique for post-pyloric feeding tube placement is limited. We conducted a meta-analysis to compare the performance of EM-guided versus endoscopic placement.

**Methods:**

We searched several databases for all randomised controlled trials evaluating the EM-guided vs. endoscopic placement of post-pyloric feeding tubes up to 28 July 2020. Primary outcome was procedure success rate. Secondary outcomes included reinsertion rate, number of attempts, placement-related complications, tube-related complications, insertion time, total procedure time, patient discomfort, recommendation scores, length of hospital stay, mortality, and total costs.

**Results:**

Four trials involving 536 patients were qualified for the final analysis. There was no difference between the two groups in procedure success rate (RR 0.97; 95% CI 0.91–1.03), reinsertion rate (RR 0.84; 95% CI 0.59–1.20), number of attempts (WMD − 0.23; 95% CI − 0.99–0.53), placement-related complications (RR 0.78; 95% CI 0.41–1.49), tube-related complications (RR 1.08; 95% CI 0.82–1.44), total procedure time (WMD − 18.09 min; 95% CI − 38.66–2.47), length of hospital stay (WMD 1.57 days; 95% CI − 0.33–3.47), ICU mortality (RR 0.80; 95% CI 0.50–1.29), in-hospital mortality (RR 0.87; 95% CI 0.59–1.28), and total costs (SMD − 1.80; 95% CI − 3.96–0.36). The EM group was associated with longer insertion time (WMD 4.3 min; 95% CI 0.2–8.39), higher patient discomfort level (WMD 1.28; 95% CI 0.46–2.1), and higher recommendation scores (WMD 1.67; 95% CI 0.24–3.10).

**Conclusions:**

No significant difference was found between the two groups in efficacy, safety, and costs. Further studies are needed to confirm our findings.

**Systematic review registration:**

PROSPERO (CRD42020172427)

**Supplementary Information:**

The online version contains supplementary material available at 10.1186/s40560-020-00506-8.

## Background

Malnutrition and inability to eat are conditions often encountered in inpatients. For such patients, enteral nutrition is considered to be superior to parenteral nutrition since it reduces complications, improves patient outcome, and is cheaper [1, 2]. It is common practice to place a post-pyloric feeding tube for enteral nutrition in patients who are intolerant of intragastric nutrition [3]. Endoscopic technique is typically used, but may require patient transportation between wards, pre-procedural fasting, and radiological confirmation of the tube’s position. Since first reported by Phang et al. in 2006 [4], electromagnetic (EM)-guided technique has been increasingly used for post-pyloric feeding tube placement. It has been suggested to be convenient and lead to a significant cost reduction. With increasing availability and familiarity with this technique, several randomised controlled trials (RCTs) [5–8] have compared EM-guided versus endoscopic (ENDO) technique. These RCTs were limited because of small sample sizes. We therefore conducted a meta-analysis to compare the performance between EM and ENDO.

## Methods

This meta-analysis follows the Preferred Reporting Items for Systematic Reviews and Meta-Analysis (PRISMA) statement [9] and was registered on PROSPERO (CRD42020172427).

### Search strategy

Two investigators (Y-W and Y-Z) independently searched MEDLINE, EMBASE, the Cochrane Library, and Google Scholar for all entries through 28 July 2020 using the following search terms: “Cortrak”, “electromagnetic”, “endoscopic”, “nasoenteral, or post-pyloric”, and “tube(s), feeding, or nutrition” (see Supplemental Digital Content 1, for exemplar PubMed search). Then, they compared their lists of potentially eligible titles and abstracts and achieved a consensus on full review.

### Study selection criteria

The following criteria were used to select studies for inclusion: (i) studies directly compared EM-guided versus endoscopic placement of post-pyloric feeding tubes; (ii) RCTs; and (iii) were English language articles. All retrospective studies, non-controlled studies, reviews, case series, abstracts, editorials, letters to editor, animal studies, duplicate studies, and studies without data on any of the primary or secondary outcomes were excluded.

### Study selection and data extraction

Decisions about study inclusion and exclusion were made independently by two investigators (Y-W and W-H). Two investigators (Z-J and W-H) extracted data independently from each study. We collected author, year of publication, country of origin, number of centres, participating operators, patient demographics, indications for enteral nutrition, and study outcomes. Any discrepancies were resolved by mutual discussion.

### Outcome measures

Our primary outcome was procedure success rate (defined as the percentage of successful tube placement in the desired location as determined). Secondary outcomes included reinsertion rate (defined as the percentage of patients undergoing reinsertion after an unsuccessful primary procedure or dislodgement/blockage of the tube), number of attempt, placement-related complications (e.g. epistaxis, gastrointestinal tract blood, and abdominal pain), tube-related complications (e.g. dislodgement, blockage, and aspiration), insertion time (defined as the time interval from insertion of the tube until fixation of the tube to the nostrils), total procedure time (including time for preparation and recovery), patient discomfort (recorded with scores in the range 0 [no complaints] to 10 [maximum complaints]), patient recommendation (recorded with scores in the range 0 [not recommended] to 10 [highly recommended]), length of hospital stay, intensive care unit (ICU) mortality, in-hospital mortality, and total costs (including the costs for tube placement procedure, complications, and therapeutic interventions).

### Validity assessment

Risk of bias assessment of RCTs was performed independently by two investigators (Y-W and Z-J) using the Cochrane Collaboration’ s tool [10]. Any disagreements were resolved by consensus after a mutual discussion. We also assessed the confidence in the estimates derived using the Grading of Recommendations Assessment, Development, and Evaluation (GRADE) approach. As all the studies included are RCTs, using the GRADE system they are considered of high quality and are downgraded to levels of moderate, low, or very low quality based on the risk of bias, indirectness, imprecision, inconsistency, and publication bias [11].

### Statistical analysis

Risk ratios (RRs) were calculated for categorical variables. Studies with no events in both arms were excluded from the meta-analysis of RRs [12]. Standard mean differences (SMDs) were calculated for continuous variables including total costs based on different monetary units. Weighted mean differences (WMDs) were calculated for the rest of continuous variables. In cases of missing data, especially for continuous variables, we estimated the mean and standard deviation from the sample size, median, range, and/or interquartile range, if available, according to the approximation method previously validated [13]. Owning to variation in study protocols and study populations, a random effects model for all analyses was used [12]. Heterogeneity among studies was assessed by calculating the *I*^2^ statistics and tested using the Cochrane Q-test [14]. Subgroup analyses were performed for all outcomes based on the following: (a) study setting (single- vs. multicentre), (b) geographical location (Asia vs. Europe), (c) the BMI level of patients with the validated method (ENDO group) (< 25 vs. > 25), (d) prior altered upper gastrointestinal anatomy (with vs. without), (e) patient population (critically ill vs. non-critically ill patients). For those subgroups with only one study included, subgroup analyses were not performed. We had planned that if enough studies (≥ 10) were included in the analysis of primary outcome, we would construct a funnel plot to evaluate publication bias [12]; otherwise, Egger’s test was applied [15]. Statistical analyses were performed using Review Manager 5.3 (The Cochrane Collaboration, The Nordic Cochrane Centre, Copenhagen, Denmark).

## Results

### Search strategy yield and study characteristics

**Fig. 1:**
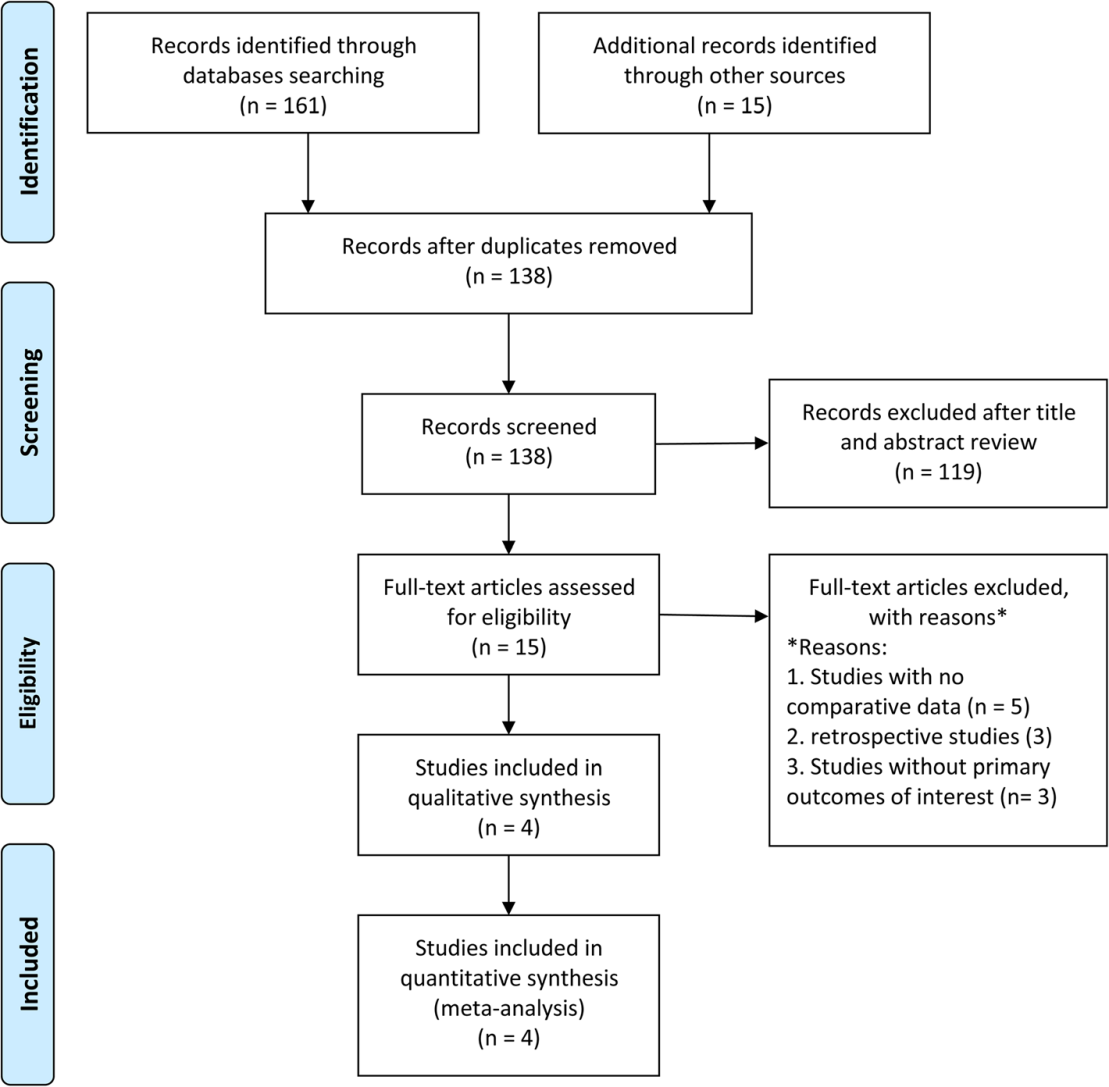
The PRISMA flow diagram of selected studies

The search identified 176 potentially relevant studies, 15 of which were examined in detail and 4 RCTs of which were included in the final analysis [15] (Fig. 1). A total of 536 patients were included, of which 287 were in the EM group and 249 in the ENDO group. Mean age was 57.1 years with a range of 51.5 to 64.6 years. The sex distribution was 53.3% male patients, with a range of 51.3 to 63.6%. Two studies were conducted in Netherlands [6, 7], one in Austria [5], and one in China [8]. Two of the studies [8] were of multicentre design (3 or 5 recruiting sites). Two studies [5, 8] exclusively evaluated critical ill patients, one [6] evaluated patients from gastrointestinal surgical wards, and the remaining one [7] included outpatients, ward patients, and critically ill patients. In the ENDO group, tube placement was performed by a gastroenterologist assisted by one or two nurses. For EM group, tube placement was performed by one nurse in two studies [6, 7], by a nutritional support team in one study [8], and the remaining one study [5] did not specify this issue. One study [6] included patients with prior altered upper gastrointestinal anatomy and the remaining three studies did not. For EM technique, pre-procedural fasting was required in one study [7], not required in one study [6], and the remaining two studies [5, 8] did not specify this issue. Conscious sedation in the EM group was not required in two studies [6, 8], but was used if indicated in the remaining two studies [5, 7]. For the ENDO group, conscious sedation was used in a large portion of patients. Details of the characteristics of included studies are given in Table 1.

**Table 1:** Characteristics of studies and patient demographics

Study (year)	Location	Setting	Number of centres	Type of patients	Indications for enteral nutrition	Sedation medication	Arms	Sample size, *n*	Age (year), mean ± SD	Sex, male, %	Body mass index (kg/m^2^), mean ± SD	Prior altered upper gastrointestinal anatomy, *n* (%)	Sedation, *n* (%)	Pre-procedural fasting	Use of prokinetic agents, *n* (%)	Operators required (*n*)
Holzinger, U et al. (2011) [5]	Austria	Single	1	ICU patients	Intolerance of intragastric enteral nutrition	NR	EM	44	55 ± 18	63.6	28 ± 7	0 (0)	39 (88.6)	NR	NR	NR
							ENDO	22	56 ± 15	36.4	29 ± 8	0 (0)	18 (81.8)	NR	0 (0)	Gastroenterologist (1), nurse (NR)
Gerritsen, A et al. (2016) [6]	Netherlands	Multicentre	5	Patients from gastrointestinal surgical wards	Postoperative gastroparesis, malnutrition, pancreatitis, ileus, and other	NR	EM	80	63.2 ± 14.4	51.3	25.6 (22.4–27.7) median (interquartile range)	14 (17.5)	0 (0)	None	49 (61.3)	Nurse (1)
							ENDO	74	64.6 ± 13.1	56.8	24.7 (22.4–26.9) median (interquartile range)	14 (18.9)	61 (82.4)	Yes	46 (62.2)	Gastroenterologist (1), nurse (1–2)
Kappelle, WFW et al. (2018) [7]	Netherlands	Multicentre	3	ICU patients and non-ICU patients	Postoperative gastroparesis,critical illness gastroparesis,pancreatitis, severe GERD, severe vomiting, low intake, and other	Propofol	EM	82	57.9 ± 16.8	53.7	NR	0 (0)	11 (12.9)	Yes	7 (9)	Nurse (1)
							ENDO	73	56.6 ± 14.3	60.3	NR	0 (0)	43 58.9)	Yes	0 (0)	Gastroenterologist (1), nurse (1–2)
Gao, XJ et al. (2018) [8]	China	Single	1	ICU patients	Intolerance of intragastric enteral nutrition	Propofol	EM	81	51.5 ± 18.3	53.1	21.2 ± 3.3	0 (0)	0 (0)	NR	81 (100)	Member of nutritional support team (1)
							ENDO	80	52.3 ± 18.2	51.3	21.6 ± 3.2	0 (0)	80 (100)	Yes	80 (100)	Gastroenterologist (1), nurse (1–2)

*RCT* randomised controlled trial, *EM* electromagnetic-guided nasoenteral feeding tube placement, *ENDO* endoscopic nasoenteral feeding tube placement, *GERD* gastroesophageal reflux disease, *ICU* intensive care unit, *NR* not reported

### Quality assessment

All of the included studies had a high risk of performance bias. This could not be avoided because the operators and patients could not be blinded to the method of examination. There was unclear risk of detection bias in all studies, as blinding of outcome assessment could only be done for subjective outcomes such as patient discomfort and patient recommendation. One study [5] had an unclear risk of selection bias owing to no reported concealment of allocations. Assessment of risk of bias is shown in Supplemental Digital Content 2.

### Primary outcome: procedure success rate

**Fig. 2:**
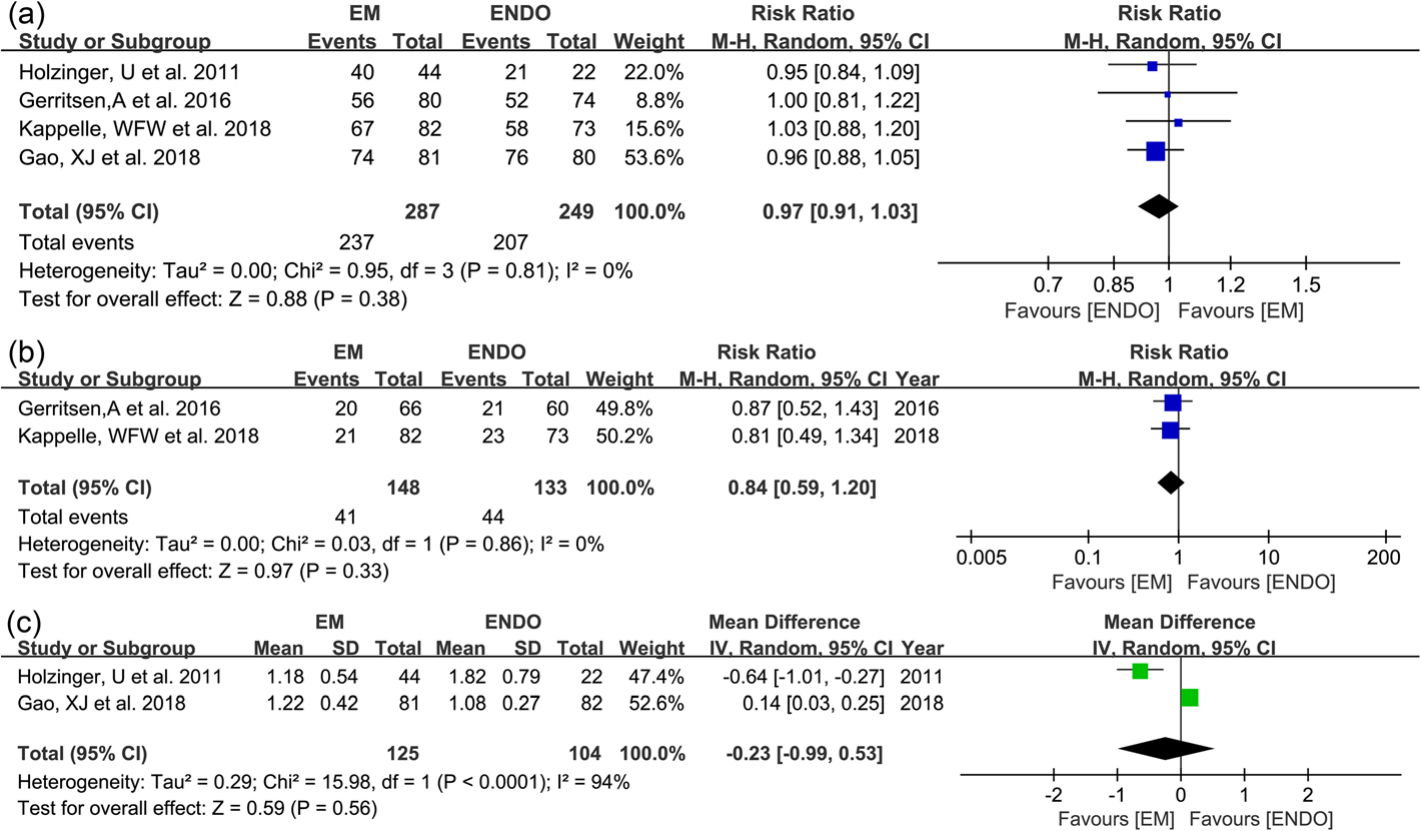
Forest plots for treatment success between EM and ENDO. **a** Procedure success rate. **b** Reinsertion rate. **c** Number of attempts

Procedure success rate was reported in all included studies (Table 2). Pooled rate for EM and ENDO was 82.6% and 83.1%, respectively. No statistical difference was observed (RR 0.97; 95% confidence interval [95% CI] 0.91–1.03; *I*^2^ = 0%; GRADE = moderate) (Fig. 2a). We did not employ funnel plot to access for publication bias as fewer than 10 studies were included. There was no evidence of publication bias by Egger’ s test for the primary outcome (*P* = 0.18).

**Table 2:** Outcomes evaluated in studies

Study (year)	Arms	Procedure success rate, *n* (%)	Position of placed tube, (*n*)	Reinsertions, *n* (%)	Number of attempts, mean ± SD	Placement-related complications, *n* (%)	Tube-related complications, *n* (%)	Insertion time (min), median (IQR)	Total procedure time (min), median (IQR)	Patient discomfort, median (IQR)	Patient recommendation, median (IQR)	Length of hospital stay (days), median (IQR)	ICU mortality, *n* (%)	In-hospital mortality, *n* (%)	Total costs, mean ± SD
Holzinger, U et al. (2011) [5]	EM	40 (90.9)	NR	NR	1.18 ± 0.54	8 (18.2)	NR	11 (6–19)	NR	NR	NR	NR	12 (27.3)	17 (38.6)	NR
	ENDO	21 (95.5)	NR	NR	1.82 ± 0.79	4 (18.2)	NR	15 (10–21)	NR	NR	NR	NR	7 (31.8)	9 (40.9)	NR
Gerritsen, A et al. (2016) [6]	EM	56 (70.9)	Horizontal duodenum (15) Ascending duodenum (20) Jejunum (20) Jejunal limb of anastomosis (3)	20 (30.3)	NR	2 (2.6)	43 (53.8)	15 (10–27)	31 (25–45)	3.9 (2.0–6.7)	8.2 (4.8–9.9)	12 (7–22)	2 (2.5)	2 (2.5)	€ 584 (504–669) Mean (95% BCaCI)
	ENDO	52 (70.3)	Horizontal duodenum (8) Ascending duodenum (18) Jejunum (18) Jejunal limb of anastomosis (8)	21 (35.0)	NR	5 (6.8)	36 (48.6)	11 (8–18)	60 (40–85)	2.0 (0.2–5.6)	5.5 (2.3–7.8)	10 (7–18)	5 (6.8)	5 (6.8)	€ 700 (585–835) mean (95% BCaCI)
Kappelle, WFW et al. (2018) [7]	EM	67 (82)	Duodenal bulb (1) Descending duodenum (1) Horizontal duodenum (6) Ascending duodenum (35) Jejunum (24)	21 (26)	NR	4 (4.9)	NR	20 (10–30)	NR	NR	4 (2–6)	NR	NR	NR	$ 522.3 ± 340.5
	ENDO	58 (73)	Duodenal bulb (4) Descending duodenum (8) Horizontal duodenum (12) Ascending duodenum (23) Jejunum (11)	23 (32)	NR	4 (5.5)	NR	10 (7–13)	NR	NR	4 (0.8–7)	NR	NR	NR	$ 631.8 ± 332.5
Gao, XJ et al. (2018) [8]	EM	74 (91.4)	Horizontal duodenum (7) Ascending duodenum (9) Jejunum (46) Ligament of Treitz (11)	NR	1.22 ± 0.42	4 (5.0)	14 (17.3)	13 ± 4 mean ± SD	18 ± 3 mean ± SD	4.3 ± 1.7 mean ± SD	7.1 ± 1.8 mean ± SD	17.6 ± 8.4 mean ± SD	14 (17.3)	20 (25)	$ 333 ± 24
	ENDO	76 (95.0)	Horizontal duodenum (5) Descending duodenum (7) Jejunum (52) Ligament of Treitz (12)	NR	1.08 ± 0.27	5 (6.3)	14 (17.5)	7 ± 2.5 mean ± SD	26 ± 6 mean ± SD	3.3 ± 1.5 mean ± SD	4.9 ± 2.4 mean ± SD	16.3 ± 7.3 mean ± SD	16 (20)	22 (27.5)	$ 461 ± 28

*BCaCI* bias-corrected and accelerated confidence interval, *EM* electromagnetic-guided placement, *ENDO* endoscopic placement, *GI* gastrointestinal tract, *IQR* interquartile range, *SD* standard deviation, *NR* not reported

### Secondary outcomes

Two studies [6, 7] with 281 patients were included to evaluate reinsertion rate. Of 148 patients undergoing EM-guided placement, reinsertion occurred in 41 patients (27.7%). As for ENDO, reinsertion occurred in 44 patients (33.1%). There was no significant difference between two groups (RR 0.84; 95% CI 0.59–1.20; *I*^2^ = 0%; GRADE = moderate) (Fig. 2b). Number of attempts was reported in 2 studies [5, 8]. No significant difference was found between two groups (1.2 vs. 1.5; WMD − 0.23; 95% CI − 0.99–0.53; *I*^2^ = 94%; GRADE = low) (Fig. 2c).

**Fig. 3:**
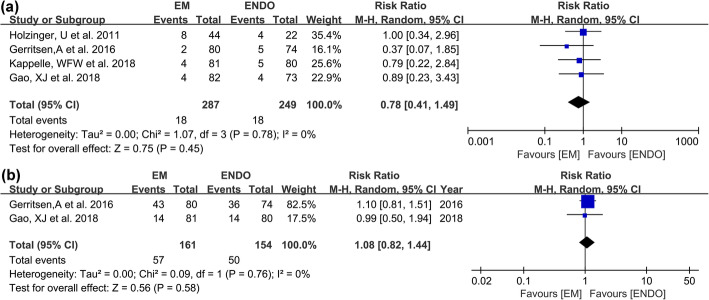
Forest plots for complications between EM and ENDO. **a** Placement-related complications. **b** Tube-related complications

All studies evaluated placement-related complications. In the EM group, placement-related complications occurred in 18 patients (6.3%), which mainly contained 10 epistaxis (3.5%), 1 gastrointestinal tract blood (0.3%), 1 hypoxia (0.3%), 1 atrial fibrillation (0.3%), and 1 abdominal pain (0.3%). As for ENDO, placement-related complications occurred in 18 patients (7.2%), which mainly contained 12 epistaxis (4.8%), 1 gastrointestinal tract blood (0.4%), and 4 hypoxias (1.6%). No significant difference was found between the two groups (RR 0.78; 95% CI 0.41–1.49; *I*^2^ = 0%; GRADE = moderate) (Fig. 3a). Two studies [6, 8] with 315 patients reported tube-related complications. In the EM group, tube-related complications occurred in 57 patients (35.4%), which mainly contained 45 dislodgements (28.0%), and 13 blockages (8.1%). As for ENDO, tube-related complications occurred in 50 patients (32.5%), which mainly contained 38 dislodgements (24.7%), 7 blockages (4.5%), and 3 aspirations (1.9%). No significant difference was found in total tube-related complications (RR 1.08; 95% CI 0.82–1.44; *I*^2^ = 0%; GRADE = moderate) (Fig. 3b). No procedure-related mortality was reported among studies.

**Fig. 4:**
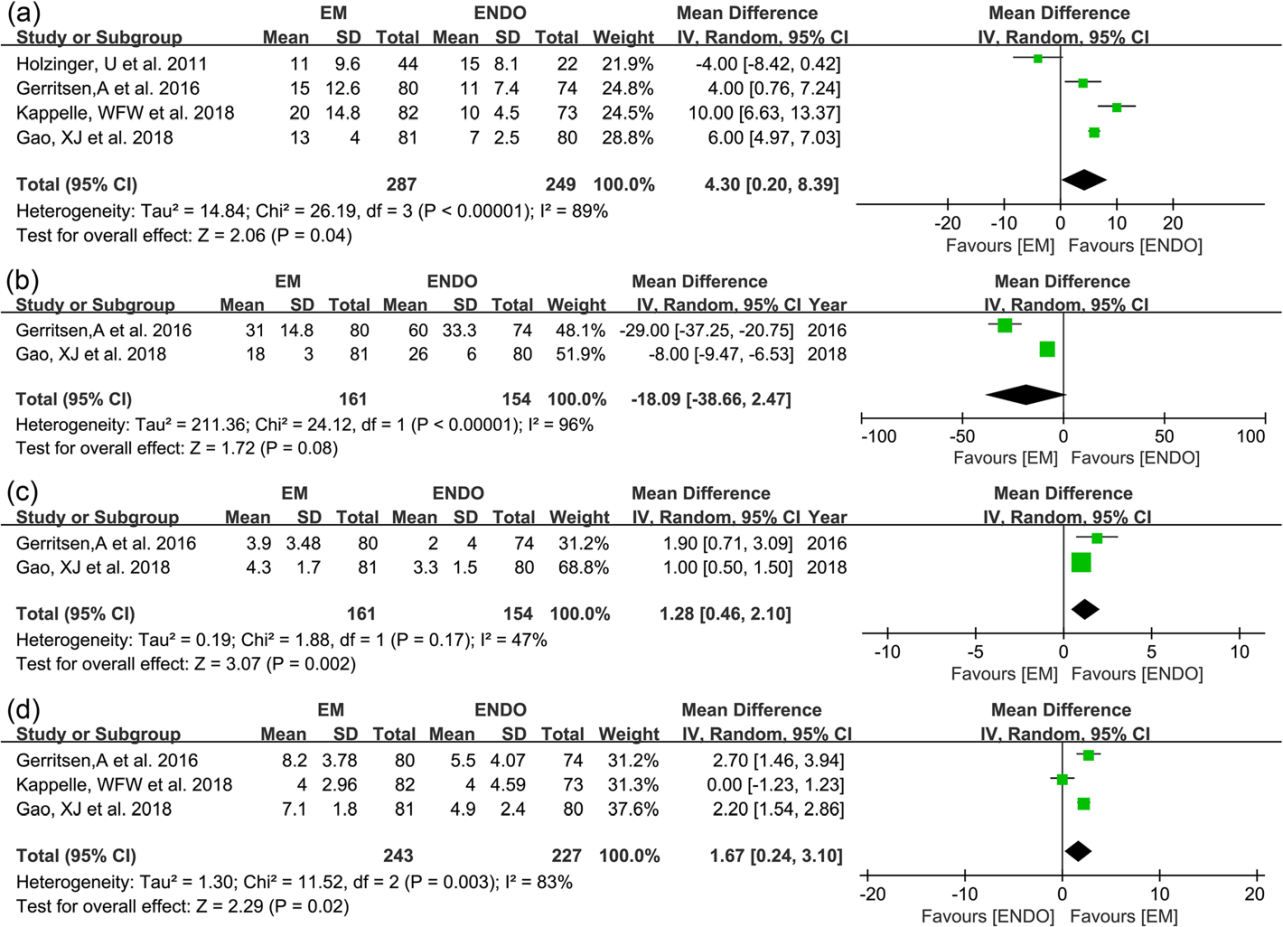
Forest plots for **a** insertion time, **b** total procedure time, **c** patient discomfort, and **d** patient recommendation between EM and ENDO

All included studies evaluated insertion time. EM was associated with longer insertion time than ENDO (14.8 min vs. 10.8 min; WMD 4.3 min; 95% CI 0.2–8.39; *I*^2^ = 89%; GRADE = low) (Fig. 4a). Total procedure time was reported in 2 studies [6, 8] (*n* = 315). No significant difference was found between two groups (24.5 min vs. 43 min; WMD − 18.09 min; 95% CI − 38.66–2.47; *I*^2^ = 96%; GRADE = low) (Fig. 4b).

Patient-assessed discomfort was reported in 2 studies [6, 8] (*n* = 315). EM showed higher discomfort level than ENDO (WMD 1.28; 95% CI 0.46–2.1; *I*^2^ = 47%; GRADE = moderate) (Fig. 4c). Patient recommendation was reported in 3 studies [6–8] (*n* = 470). The EM group received higher recommendation scores than the ENDO group (WMD 1.67; 95% CI 0.24–3.10; *I*^2^ = 83%; GRADE = low) (Fig. 4d).

**Fig. 5:**
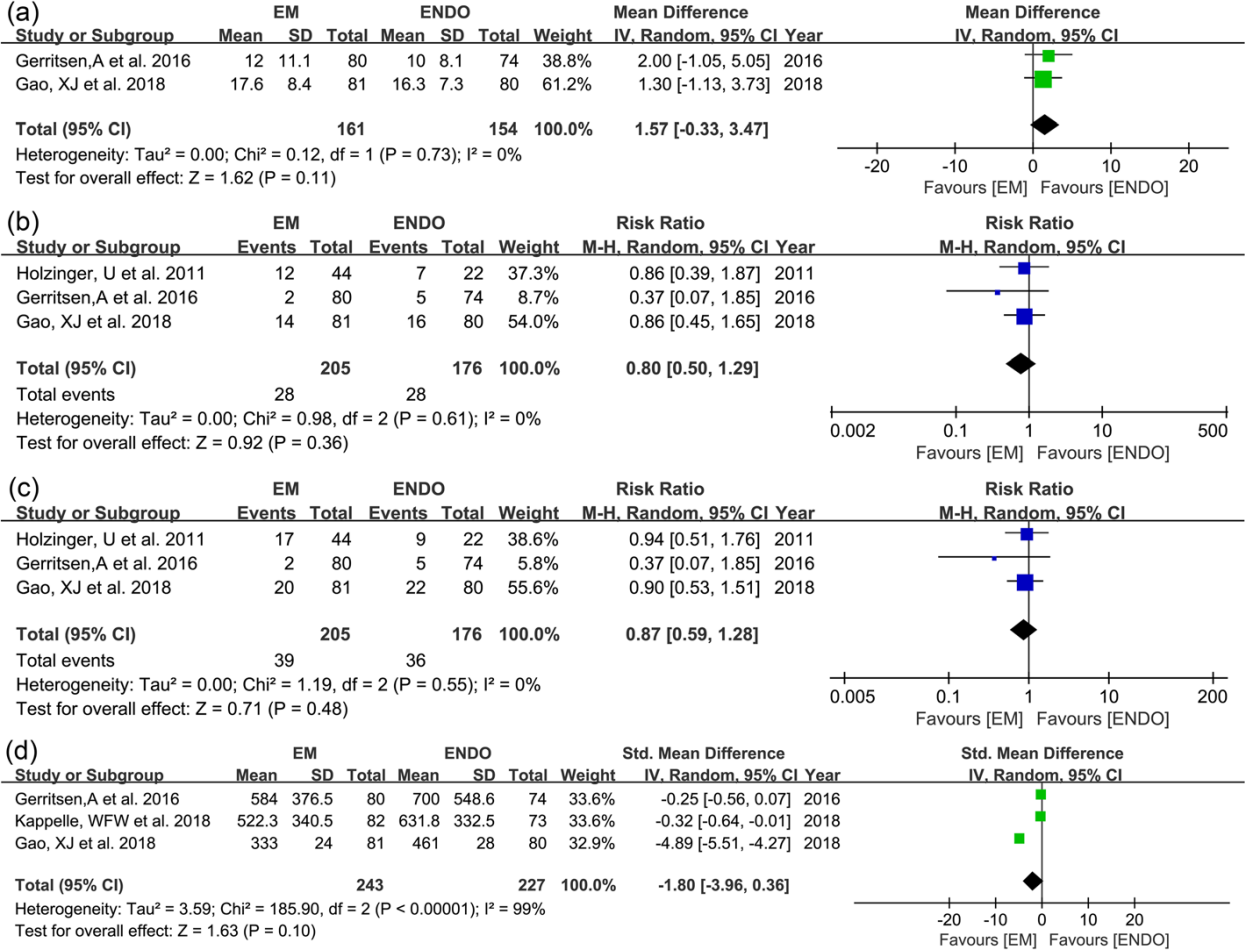
Forest plots for **a** length of hospital stay, **b** ICU mortality, **c** in-hospital mortality, and **d** total costs of the feeding tube placement between EM and ENDO

Length of hospital stay was reported in 2 studies [6–8] (*n* = 315). No significant difference was found between the 2 groups (14.8 days vs. 13.2 days; WMD 1.57 days; 95% CI − 0.33–3.47; *I*^2^ = 0%; GRADE = moderate) (Fig. 5a). Mortality was reported in 3 studies [5, 6, 8] (*n* = 381). There was no difference between the two groups in ICU mortality (RR 0.80; 95% CI 0.50–1.29; *I*^2^ = 0%; GRADE = moderate) (Fig. 5b) and in-hospital mortality (RR 0.87; 95% CI 0.59–1.28; *I*^2^ = 0%) (Fig. 5c).

Total costs were provided in 3 studies [6–8] (*n* = 470). Currency units were variably used euro and dollar. No significant difference was found between the two groups (SMD − 1.80; 95% CI − 3.96–0.36; *I*^2^ = 99%; GRADE = low) (Fig. 5d). Table 3 shows the quality of evidence using the GRADE assessment tool which is detailed for each of the outcomes.

**Table 3:** GRADE analysis and assessment of quality of evidence

Quality assessment							Summary of findings				
							No of patients		Effect		Quality
No of studies	Design	Limitations	Inconsistency	Indirectness	Imprecision	Other considerations	EM	ENDO	Relative (95% CI)	Absolute	
**Procedure success rate**											
4	randomised trials	serious^a^	no serious inconsistency	no serious indirectness	no serious imprecision	none	237/287 (82.6%)	207/249 (83.1%)	RR 0.97 (0.91 to 1.03)	25 fewer per 1000 (from 75 fewer to 25 more)	⊕⊕⊕○ MODERATE
								87.2%		26 fewer per 1000 (from 78 fewer to 26 more)	
**Need for reinsertion**											
2	randomised trials	serious^a^	no serious inconsistency	no serious indirectness	no serious imprecision	none	41/148 (27.7%)	44/133 (33.1%)	RR 0.84 (0.59 to 1.2)	53 fewer per 1000 (from 136 fewer to 66 more)	⊕⊕⊕○ MODERATE
								33.3%		53 fewer per 1000 (from 137 fewer to 67 more)	
**Number of attempts**											
2	randomised trials	serious^a^	serious^b^	no serious indirectness	no serious imprecision	none	125	104	-	MD 0.23 lower (0.99 lower to 0.53 higher)	⊕⊕○○ LOW
**Placement-related complications**											
4	randomised trials	serious^a^	no serious inconsistency	no serious indirectness	no serious imprecision	none	18/287 (6.3%)	18/249 (7.2%)	RR 0.78 (0.41 to 1.49)	16 fewer per 1000 (from 43 fewer to 35 more)	⊕⊕⊕○ MODERATE
								6.5%		14 fewer per 1000 (from 38 fewer to 32 more)	
**Tube-related complications**											
2	randomised trials	serious^a^	no serious inconsistency	no serious indirectness	no serious imprecision	none	57/161 (35.4%)	50/154 (32.5%)	RR 1.08 (0.82 to 1.44)	26 more per 1000 (from 58 fewer to 143 more)	⊕⊕⊕○ MODERATE
								33.1%		26 more per 1000 (from 60 fewer to 146 more)	
**Insertion time**											
4	randomised trials	serious^a^	serious^b^	no serious indirectness	no serious imprecision	none	287	249	-	MD 4.3 higher (0.2 to 8.39 higher)	⊕⊕○○ LOW
**Total procedure time**											
2	randomised trials	serious^a^	serious^b^	no serious indirectness	no serious imprecision	none	161	154	-	MD 18.09 lower (38.66 lower to 2.47 higher)	⊕⊕○○ LOW
**Patient discomfort**											
2	randomised trials	serious^a^	no serious inconsistency	no serious indirectness	no serious imprecision	none	161	154	-	MD 1.28 higher (0.46 to 2.1 higher)	⊕⊕⊕○ MODERATE
**Patient recommendation**											
3	randomised trials	serious^a^	serious^b^	no serious indirectness	no serious imprecision	none	243	227	-	MD 1.67 higher (0.24 to 3.1 higher)	⊕⊕○○ LOW
**Length of hospital stay**											
2	randomised trials	serious^a^	no serious inconsistency	no serious indirectness	no serious imprecision	none	161	154	-	MD 1.57 higher (0.33 lower to 3.47 higher)	⊕⊕⊕○ MODERATE
**ICU mortality**											
3	randomised trials	serious^a^	no serious inconsistency	no serious indirectness	no serious imprecision	none	28/205 (13.7%)	28/176 (15.9%)	RR 0.8 (0.5 to 1.29)	32 fewer per 1000 (from 80 fewer to 46 more)	⊕⊕⊕○ MODERATE
								20%		40 fewer per 1000 (from 100 fewer to 58 more)	
**In-hospital mortality**											
3	randomised trials	serious^a^	no serious inconsistency	no serious indirectness	no serious imprecision	none	39/205 (19%)	36/176 (20.5%)	RR 0.87 (0.59 to 1.28)	27 fewer per 1000 (from 84 fewer to 57 more)	⊕⊕⊕○ MODERATE
								27.5%		36 fewer per 1000 (from 113 fewer to 77 more)	
**Total costs**											
3	randomised trials	serious^a^	serious^b^	no serious indirectness	no serious imprecision	none	243	227	-	SMD 1.8 lower (3.96 lower to 0.36 higher)	⊕⊕○○ LOW

^a^Blinding of participants and personnel are impossible in all RCTs

^b^Statistical heterogeneity between RCTs

### Subgroup analyses

Subgroup analyses were performed for all outcomes (Supplemental Digital Content 3). EM compared with ENDO was associated with lower total cost when only evaluating multicentre RCTs (SMD − 0.29; 95% CI − 0.51 to − 0.06; *I*^2^ = 0%). Other subgroup analysis results were accordant to the main analyses. Heterogeneity was reduced when subgroup analyses for total costs and insertion time was performed using level of BMI < 25 as a modifier. The other heterogeneity could not be explained by these variables with the available data.

## Discussion

The conventional methods for the placement of post-pyloric feeding tubes include blind, fluoroscopic, and endoscopic methods [16, 17]. The gold standard is the endoscopic technique, which has success rates above 90% [18, 19]. Bedside EM-guided tube placement can be performed in recent years [20]. This has several potential advantages compared with endoscopic placement because only one trained nurse and less equipment is needed [21, 22]. EM and ENDO techniques have been compared in only one systematic review until now [23]. That review by Gerritsen et al. involving only one relevant RCT (66 patients) concluded that the efficacy and safety of the two techniques did not differ significantly, but EM offered advantages in logistics. In view of 3 new RCTs published, we have attempted to pool the evidence to further evaluate the performance of EM vs. ENDO.

Regarding treatment success, our meta-analysis did not demonstrated difference in procedure success rate, reinsertion rate, and number of attempts. These results were robust across the subgroup analyses. Our procedure success rates of the two groups (82.6% vs. 83.1%) were lower than those reported in the previous systematic review (85% vs. 89%), and our reinsertion rates (27.7% vs. 33.1%) were higher than theirs (21% vs. 16%). The reasonable explanation may be that our review included many gastrointestinal disease patients, with an altered upper gastrointestinal anatomy, which may hamper tube placement.

With respect to safety, our meta-analysis did not provide evidence that difference existed in complication rates, length of hospital stay, ICU mortality, and in-hospital mortality. Subgroup analysis results were consistent with main analyses in these outcomes. Average placement- and tube-related complication rates for EM that have been reported in previous review was 0.4% and 15%, our finding (6.3% and 35.4%) was significantly higher than theirs. The most common complications in our review were epistaxis and dislodgment/blockage of the tube. However, undetected statistical difference in complications may be attributed to low incidence and insufficient sample size. More large-sample RCTs are required to investigate these outcomes in greater detail. It is worth noting that, unlike EM, sedation is often required during endoscopic technique. Sedation is associated with a small risk of cardiopulmonary adverse events, e.g. hypoxia [24]. This could account for the difference in hypoxia incidence between the two techniques (0.3% vs. 1.6%).

Although EM was more time consuming for insertion, the fact that EM technique does not require sedation and patient transportation resulted in comparable total time compared with endoscopy. Another advantage of EM is that in case of partial migration of the tube, repositioning of the tube can be done by reinserting the stylet through the tube at the bedside, whereas repositioning by endoscopy would require removal of the tube and repetition of the entire procedure (including sedation). Regarding patient-reported outcomes, patients reported more discomfort during EM-guided placement than during endoscopy. This finding is probably related to the large differences in the use of conscious sedation between the two groups. On the other hand, recommendation scores were significantly higher in the EM group, presumably because the discomfort is not bothersome enough to advise others not to undergo the EM procedure. This supports the hypothesis that EM is a more patient-friendly approach.

Finally, as for total costs, we found absence of evidence for difference between the two groups. The reduction in the use of hospital resources (e.g. personnel, patient transportation) and the absence of the need for sedation and radiographic confirmation can lead to a reduction in costs. Therefore, the cost difference between EM and ENDO we found may be an underestimation of the true cost difference. This could also be evidenced in subgroup analyses: lower total cost with EM was noted, when only evaluating multicentre RCTs. More well-designed multicentre RCTs are therefore warranted.

Our meta-analysis has several strengths. First, we conducted rigorous search of the RCTs and added 3 additional studies that the previous systematic review did not include. Second, comprehensive subgroup analyses were performed. Last, we assessed multiple clinically relevant outcomes. Because critically ill and non-critically ill patients were both included, the results of our review are probably generalizable to the overall hospital population. However, limitations are present and are as follows. The number of included studies is relatively small. Variation among studies was observed in study design, introducing heterogeneity. Data on patients with prior altered upper gastrointestinal anatomy was not enough for subgroup analyses.

## Conclusions

Based on the currently available literature, our meta-analysis does not demonstrate the difference between EM and ENDO in safety, efficacy, and total cost. Further studies are needed to confirm our findings.

## Supplementary Information

**Additional file 1:** **Supplemental Digital Content 1**. PubMed search strategy. **Supplemental Digital Content 2**. Assessment of risk of bias. Green denotes low risk of bias, red indicates high risk of bias, and yellow represents unclear risk of bias. **Supplemental Digital Content 3**. Subgroup analyses (EM vs. ENDO).

## Data Availability

All the data supporting the conclusions of this article are included within the article.

## Abbreviations

EM: Electromagnetic
ENDO: Endoscopic
PRISMA: the Preferred Reporting Items for Systematic Reviews and Meta-Analysis
RCT: Randomised clinical trial
RR: Risk ratio
SD: Standard deviation
SMD: Standard mean difference
WMD: Weighted mean difference
