# Supplementary material for: Electromagnetic-guided versus endoscopic placement of post-pyloric feeding tubes: a systematic review and meta-analysis of randomised controlled trials
Source: J Intensive Care. 2020 Dec 10;8:92. doi: 10.1186/s40560-020-00506-8 (PMC7727211; doi:10.1186/s40560-020-00506-8)
Supplement: Supplementary file 1 — Additional file 1: Supplemental Digital Content 1. PubMed search strategy. Supplemental Digital Content 2. Assessment of risk of bias. Green denotes low risk of bias, red indicates high risk of bias, and yellow represents unclear risk of bias. Supplemental Digital Content 3. Subgroup analyses (EM vs. ENDO). [file 40560_2020_506_MOESM1_ESM.docx]

Supplemental Digital Content 1. **PubMed-MEDLINE search strategy**

up to 28 July 2020.

#1 electromagn*[tiab] OR cortrak[tiab]

#2 endoscop*[tiab]

#3 #1 AND #2

#4 nasoenteral[tiab] OR nasojejunal[tiab] OR nasoduodenal[tiab] OR postpyloric[tiab] OR post-pyloric[tiab] OR nasointestinal[tiab] OR nasoenteric[tiab] #5 "randomized controlled trial"[pt] OR "controlled clinical trial"[pt] OR "randomized"[tiab] OR "randomised"[tiab] OR "randomly"[tiab]

#6 #3 AND #4 AND #5

**Supplemental Digital Content 2.** Assessment of risk of bias. Green denotes low risk of bias, red indicates high risk of bias, and yellow represents unclear risk of bias.


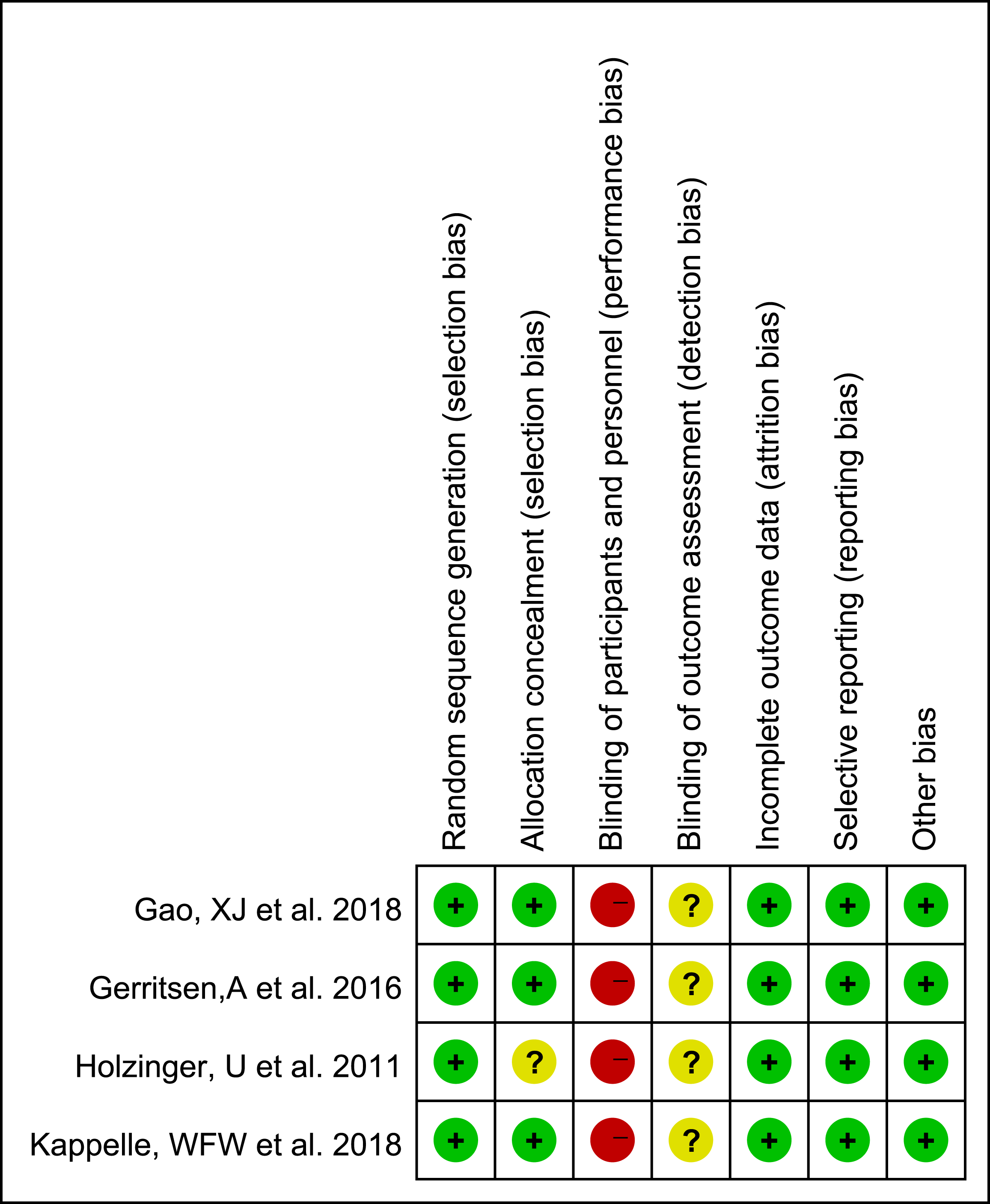


| **Supplemental Digital Content 3. Subgroup analyses (EM vs. ENDO)** | | | | |
| --- | --- | --- | --- | --- |
|  | **Number of studies** | **Patients, n** | **RR [95% CI], WMD [95% CI], or SMD [95% CI]** | **I^2^** |
| *Procedure success rate^†^* |  |  |  |  |
| Single-center^5, 8^ | 2 | 227 | 0.96 [0.89, 1.03] | 0% |
| Multi-center^6, 7^ | 2 | 309 | 1.02 [0.90, 1.15] | 0% |
| Europe ^5-7^ | 3 | 375 | 0.99 [0.90, 1.08] | 0% |
| Level of BMI<25^6, 8^ | 2 | 315 | 0.97 [0.89, 1.04] | 0% |
| Without prior altered upper gastrointestinal anatomy ^5, 7, 8^ | 3 | 382 | 0.97 [0.91, 1.03] | 0% |
| Critically ill patients^5, 8^ | 2 | 227 | 0.96 [0.89, 1.03] | 0% |
| *Placement-related complications^†^* |  |  |  |  |
| Single-center^5, 8^ | 2 | 227 | 0.96 [0.41, 2.23] | 0% |
| Multi-center^6, 7^ | 2 | 309 | 0.59 [0.22, 1.60] | 0% |
| Europe ^5-7^ | 3 | 375 | 0.75 [0.36, 1.57] | 0% |
| Level of BMI<25^6, 8^ | 2 | 315 | 0.62 [0.22, 1.74] | 0% |
| Without prior altered upper gastrointestinal anatomy ^5, 7, 8^ | 3 | 382 | 0.90 [0.45, 1.83] | 0% |
| Critically ill patients^5, 8^ | 2 | 227 | 0.96 [0.41, 2.23] | 0% |
| *Patient recommendation^‡^* |  |  |  |  |
| Multi-center^6, 7^ | 2 | 309 | 1.35 [-1.30, 3.99] | 89% |
| Level of BMI<25^6, 8^ | 2 | 315 | **2.31 [1.73, 2.89]**^¶^ | **0%** |
| *Duration of total placement procedure^‡^* |  |  |  |  |
| Level of BMI<25^6, 8^ | 2 | 315 | -18.09 [-38.66, 2.47] | 96% |
| *Insertion time^‡^* | | | | |
| Single-center^5, 8^ | 2 | 227 | 1.24 [-8.55, 11.03] | 95% |
| Multi-center^6, 7^ | 2 | 309 | **6.98 [1.10, 12.86]**^¶^ | 84% |
| Europe ^5-7^ | 3 | 375 | 3.46 [-3.90, 10.81] | 92% |
| Level of BMI<25^6, 8^ | 2 | 315 | **5.61 [4.06, 7.16]^¶^** | 25% |
| Without prior altered upper gastrointestinal anatomy ^5, 7, 8^ | 3 | 382 | 4.27 [-1.55, 10.09] | 92% |
| Critically ill patients^5, 8^ | 2 | 227 | 1.24 [-8.55, 11.03] | 95% |
| **Supplemental Digital Content 3. Continued.** | | | | |
|  | **Number of studies** | **Patients, n** | **RR [95% CI], WMD [95% CI], or SMD [95% CI]** | **I^2^** |
| *Total costs*^§^ | | | | |
| Multi-center^6, 7^ | 2 | 309 | **-0.29 [-0.51, -0.06]^¶^** | 0% |
| Level of BMI<25^6, 8^ | 2 | 315 | -2.56 [-7.11, 1.99] | 99% |
| *Length of hospital stay^‡^* | | | | |
| Level of BMI<25^6, 8^ | 2 | 315 | 1.57 [-0.33, 3.47] | 0% |
| *In-hospital mortality^†^* | | | | |
| Single-center^5, 8^ | 2 | 227 | 0.88 [0.49, 1.57] | 0% |
| Level of BMI<25^6, 8^ | 2 | 315 | 0.76 [0.39, 1.45] | 0% |
| Critically ill patients^5, 8^ | 2 | 227 | 0.88 [0.49, 1.57] | 0% |
| *ICU mortality^†^* | | | | |
| Single-center^5, 8^ | 2 | 227 | 0.82 [0.43, 1.58] | 0% |
| Level of BMI<25^6, 8^ | 2 | 315 | 0.71 [0.35, 1.46] | 0% |
| Critically ill patients^5, 8^ | 2 | 227 | 0.82 [0.43, 1.58] | 0% |
| CI, confidence interval; RR, risk ratio; WMD, weighted mean difference; SMD, standard mean difference; EM, electromagnetic-guided placement of nasoenteral feeding tubes; ENDO, endoscopic placement of nasoenteral feeding tubes; BMI: body mass index (kg/m^2^).  ^†^RR; ^‡^WMD; ^§^SMD; ^¶^Bold values are statistically significant. | | | | |
